# Low-cost cross-taxon enrichment of mitochondrial DNA using in-house synthesised RNA probes

**DOI:** 10.1371/journal.pone.0209499

**Published:** 2019-02-04

**Authors:** Stephen M. Richards, Nelli Hovhannisyan, Matthew Gilliham, Joshua Ingram, Birgitte Skadhauge, Holly Heiniger, Bastien Llamas, Kieren J. Mitchell, Julie Meachen, Geoffrey B. Fincher, Jeremy J. Austin, Alan Cooper

**Affiliations:** 1 Australian Centre for Ancient DNA, University of Adelaide, Adelaide, Australia; 2 Yerevan State University, Yerevan, Armenia; 3 ARC Centre of Excellence in Plant Energy Biology, Waite Research Institute, University of Adelaide, Adelaide, Australia; 4 Carlsberg Research Laboratory, Copenhagen, Denmark; 5 Des Moines University, Des Moines, Iowa, United States of America; 6 ARC Centre of Excellence in Cell Walls, Waite Research Institute, University of Adelaide, Adelaide, Australia; University of Florence, ITALY

## Abstract

Hybridization capture with in-solution oligonucleotide probes has quickly become the preferred method for enriching specific DNA loci from degraded or ancient samples prior to high-throughput sequencing (HTS). Several companies synthesize sets of probes for in-solution hybridization capture, but these commercial reagents are usually expensive. Methods for economical in-house probe synthesis have been described, but they do not directly address one of the major advantages of commercially synthesised probes: that probe sequences matching many species can be synthesised in parallel and pooled. The ability to make “phylogenetically diverse” probes increases the cost-effectiveness of commercial probe sets, as they can be used across multiple projects (or for projects involving multiple species). However, it is labour-intensive to replicate this with in-house methods, as template molecules must first be generated for each species of interest. While it has been observed that probes can be used to enrich for phylogenetically distant targets, the ability of this effect to compensate for the lack of phylogenetically diverse probes in in-house synthesised probe sets has not been tested. In this study, we present a refined protocol for in-house RNA probe synthesis and evaluated the ability of probes generated using this method from a single species to successfully enrich for the target locus in phylogenetically distant species. We demonstrated that probes synthesized using long-range PCR products from a placental mammal mitochondrion (*Bison* spp.) could be used to enrich for mitochondrial DNA in birds and marsupials (but not plants). Importantly, our results were obtained for approximately a third of the cost of similar commercially available reagents.

## Introduction

Hybridization capture and high-throughput sequencing (HTS) have become a powerful combination of tools for sequencing genomic targets from degraded samples such as ancient DNA (aDNA) [1,2]. Hybridization capture enriches loci of interest, which lowers the sequencing effort needed and thus reduces the costs of a study. Hybridization capture uses complementary oligonucleotide probes to bind and immobilize target DNA allowing unwanted sequences to be washed away and can be performed with the probes in-solution or attached to a solid support. The combination of hybridization capture and HTS has allowed large genomic targets to be recovered from sub-fossil specimens including a whole chromosome [3], millions of single nucleotide polymorphisms [2], and nuclear genomes [4]. However, commercial probe sets for enriching large genomic targets require considerable expenditure to purchase, and may be excessive for many projects (such as those targeting only mitochondrial genomes). While a few companies (e.g. Arbor Biosciences) will synthesize probes for smaller targets, these reagents can be expensive especially for small-scale experiments.

To minimise the costs of hybridisation enrichment experiments for studies of small targets, attempts have been made to develop in-house probe production methods [5]. While this partially reduces the necessary expenditure, one of the advantages of commercially synthesised probe sets is that probe sequences matching many different species can be included, creating a “phylogenetically diverse” set of probes. For example, a single phylogenetically diverse probe set synthesised by Arbor Biosciences has been used to enrich mtDNA from a range of extinct bird species, including: elephant birds [6], parrots [7], owls [8], ducks [9], and ravens [10]. Replicating a similarly diverse set of probes in-house would be extremely labour intensive, potentially limiting the cost-effectiveness of in-house approaches versus commercial options. However, previous studies have reported successful hybridization capture of targets from taxa phylogenetically distant from the probe sequences [11–13], though these studies used commercially synthesised probes. If in-house synthesised probes can also be used to enrich for phylogenetically distant targets, then phylogenetically diverse probe sets may be unnecessary, making in-house probe sets much more appealing and economically viable.

**Fig 1:**
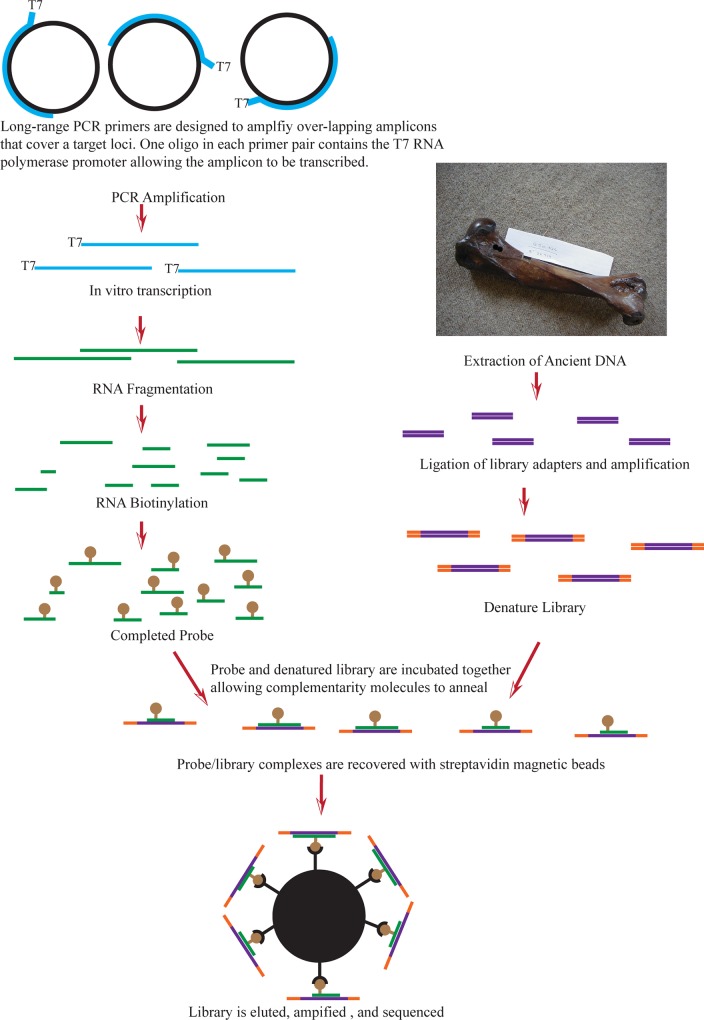
Schematic of probe synthesis and hybridization capture.

There is clearly a need for low-cost methods to produce probes for modestly sized targets (e.g. mitogenomes ≈ 16,000 bp and chloroplast genomes ≈ 150,000 bp) that will work on a diverse array of taxa. To meet this need, in the present study we present and evaluate a protocol to generate RNA probes for the enrichment of moderately sized targets (Fig 1), including an assessment of the impacts of wash stringency on capture efficiency. Previously, probes synthesized with a similar protocol have been used to enrich whole mitochondrial genomes (mitogenomes) from Pleistocene bison [14] and pre-Columbian Native Americans [15]. However, the primary goal of the current study was to determine if probes synthesized from one species could successfully enrich target DNA from phylogenetically distant taxa. First, we verified the protocol by enriching for northern hairy-nosed wombat mitochondrial DNA (mtDNA) from museum specimens using probes produced from modern northern hairy-nosed wombat tissue. Then, to test the limits of cross-taxon enrichment, probes synthesized from modern bison mtDNA were used to enrich mtDNA from museum and sub-fossil specimens of divergent taxa, which included eutherian steppe bison (extinct) and bighorn sheep, marsupial thylacine (extinct), avian emu, and even broomcorn millet. Finally, we performed a cost-analysis of our in-house probes and equivalent commercial reagents.

## Methods

### Modern DNA for probe synthesis

Modern DNA for long-range PCR of mitogenomes came from the following sources: a northern hairy-nosed wombat ear punch [16], and blood samples from American bison (*Bison bison*) and European bison (*Bison bonasus)*. DNA was isolated using a Qiagen DNeasy Blood & Tissue Kit following the manufacturer’s instructions.

### Long-range amplicons for molecular probe synthesis

Long-range PCR primers (Table 1) were designed to amplify overlapping amplicons of the entire mitochondrial genome of the American bison (GenBank accession number NC_012346.1; total length = 16319 bp) or nucleotides 1 to15420 of the northern hairy-nosed wombat mitochondrial genome (GenBank accession number KJ868118.1; total length = 17028 bp), which excluded the D-loop because of the difficulty in designing primers to amplify over this region. The T7 RNA promoter sequence was attached to the 5’ end of one primer in each primer pair to allow *in vitro* transcription of the long-range amplicon.

**Table 1:** Long-range PCR primers.

Primer Name	Amplicon Name	Primer Sequence (‘5-‘3)	Amplicon Length (bp)
	**Wombat**		
**pheF2**	**wombat-mt1**	**AATTGTAATACGACTCACTATAGGG**AAAGCAAAGCACTGAAAATGC	**5277**
**Mars_5295_tAsnR**		GGYGCTTAGCTGTTAACTAAG	
**Mars_5156_tTrpF**	**wombat-mt2**	**AATTGTAATACGACTCACTATAGGG**AGACCAAAGGCCTTCAAAGCC	**6662**
**LEU2**		GTTGCACCAAAGTTTTTGGTTCCTAAGACC	
**Mars_10916_ND4F**	**wombat-mt3**	**AATTGTAATACGACTCACTATAGGG**CAYGTAGAAGCWCCMATCGCAGG	**4567**
**ProR2fix**		AGAATRTCAGCTTTGGGTGTTGATGG	
	**Bison**		
**M856-Bovid_mt1_T7_fwd**	**bovid-mt1**	**AATTGTAATACGACTCACTATAGGG**TGACCGTGCAAAGGTAGCAT	**6616**
**M857-Bovid_mt1_rev**		AGCATGAGTTTGGTGTGTCA	
**M858-Bovid_mt2_T7_fwd**	**bovid-mt2**	**AATTGTAATACGACTCACTATAGGG**TCATTCACACCAACAACACAAC	**5946**
**M859-Bovid_mt2_rev**		TTGTAGTTGAATGACAACGATGG	
**M862-Bovid_mt3_fwd**	**bovid-mt3**	CCCTCTGCAATAGAAGGCCC	**6353**
**M863-Bovid_mt3_T7_rev**		**AATTGTAATACGACTCACTATAGGG**GATAGCGGTTGCACCATCAG	

Primer sets to amplify a partial mitogenome of the northern hairy-nosed wombat and complete mitogenome of modern bison. The T7 RNA polymerase promoter is attached to the 5’ end of one primer of each primer pair to allow transcription of the amplicon for probe synthesis. Bold nucleotides = T7 RNA polymerase promoter sequence

Amplifications were performed in PCRs using the Expand Long Range dNTPack Version 7 (Roche). Each PCR contained: 1x Expand Long Range Buffer 2, 0.5 mM dNTPs, 0.3 μM each of forward and reverse primers, 20 ng DNA template, 3.5 U Expand Long Range Polymerase, and H_2_O to 50 μL. To increase the diversity of the sequences in the bison probe pool we attempted to generate long-range amplicons from both modern European and American bison. For the bison primers, parallel PCRs were performed with one set of amplifications containing DNA from American bison and the other set containing DNA from European bison.

All long-range PCRs were performed in a heated-lid thermal cycler programmed as follows: initial denaturation at 94°C for 2:00 min; 10 cycles of 94°C for 10 sec, 55°C for 15 sec and 68°C for 7:00 min; 25 cycles of 94°C for 10 sec, 55° for 15 sec, and 68°C for 7:00 min + 20 sec/cycle; and a final elongation at 68°C for 5:00 min. All wombat long-range amplicons were successfully amplified and pooled together in equimolar concentrations. For the bison PCRs, the bovid-mt1 and bovid-mt2 amplicons could not be amplified from the modern European bison DNA. Consequently, the bison long-range amplicons were combined in the following ratios: 1 mole bovid-mt1 from American bison, 1 mole bovid-mt2 from American bison, 0.5 mole bovid-mt3 from American bison, 0.5 mole bovid-mt3 from European bison. Pools of long-range amplicons were purified with 1x volume SpeedBeads (GE Healthcare) [17], quantified with a NanoDrop spectrophotometer (ThermoFisher), and visualized with GelRed (Biotium) staining after electrophoresis on a 2% agarose gel.

### In vitro transcription of long-range amplicons

Each amplicon pool was transcribed in several *in vitro* transcription reactions using a HiScribe T7 High Yield RNA Synthesis Kit (New England Biolabs) following the provided protocol except that the incubation was carried out overnight. All transcription reactions were further incubated with 1x DNase Buffer and 4 U RNase free DNase I at 37°C for 15 minutes to remove the amplicon template. The DNase I was inactivated by adding 6 μL 0.5 M EDTA and heating at 75°C for 10 min. The RNA was then precipitated with ethanol, sodium acetate, and glycogen [18].

### Fragmentation of RNA

The precipitated RNA was suspended in nuclease free H_2_O and quantified with a NanoDrop spectrophotometer. Fragmentation of the RNA was performed by incubation in 1x NEBNext RNA Fragmentation Buffer (New England Biolabs) with heating at 94° C for 10 min. The reaction was stopped with the addition of 1x NEBNext RNA Fragmentation Stop Solution and the RNA purified with a RNeasy MinElute Cleanup kit (Qiagen) following the manufacturer's instructions.

### Biotinylation of fragmented RNA

Fragmented RNA was photolabelled with biotin using the EZ-Link Psoralen-PEG3-Biotin reagent (ThermoFisher) and the manufacturer's instructions. The labelled RNA was purified with a RNeasy MinElute Cleanup kit, quantified with a Qubit RNA BR assay (ThermoFisher), and separated into 125 ng aliquots for use in single hybridization capture reactions.

### Samples for hybridization capture

Libraries from previous studies were selected for capture with our in-house probes. Ten historic northern hairy-nosed wombat libraries made from bone/teeth samples (Queensland Museum and Museum Victoria) were selected for enrichment of partial mitochondrial genomes (Table 2). To explore the taxonomic range of hybridization capture by bison probes, samples of ancient bison, bighorn sheep, and a museum specimen thylacine (Tasmanian Museum and Art Gallery) were selected for study. A historic broomcorn millet library was also chosen for enrichment with bison probes (Table 2). The ages of the wombat and thylacine samples were estimated from collection dates while the age of the bighorn sheep sample was estimated from the stratigraphic position in which the bone was found. All other samples were carbon dated at the Oxford Radiocarbon Accelerator Unit. No permits were required for the animals native to Australia. Import permits for the samples from outside Australia were obtained in accordance with Australian Government Quarantine Act 1908 Section 13(2AA). A permit to perform research on the bighorn sheep tooth was obtained from the Bureau of Land Management (USA) and permits were not required for the scientific investigation of the remaining samples. All samples are publicly available except the broomcorn millet grain, which was destroyed in this study during the extraction of aDNA. The holding institutions and additional details on the samples is given in S1 Table. The precautions taken for working with aDNA and extraction methods are given in S1 Supplemental Methods.

**Table 2:** Samples.

ACAD Number	Scientific name	Common Name	Country Origin	Location Found	Tissue	Age—Years	Divergence Time From *Bison bison/ Bison bonasus*
**3133A**	*Bison priscus*	Steppe bison	Yukon Territory, Canada	Irish Gulch	Astragalus bone	**26360 ± 220 BP**	Unknown
**18154A**	*Ovis canadensis*	Bighorn sheep	WY, USA	Natural Trap Cave	Tooth	12,000 to 20,000	24.6 MYA
**9743A**	*Thylacinus cynocephalus*	Thylacine	Tas, Australia	Ouse, Central Highlands	Drilled tooth and bone power, inner jaw	≈ 145	159 MYA
**18220A**	*Dromaius novaehollandiae*	Emu	Tas, Australia	Mole Creek	Bone	**1010 ± 30 BP**	320 MYA
**11295A**	*Panicum miliaceum*	Broomcorn millet	Vayots Dazor, Armenia	Areni-1 Cave	Seed	**1118 ± 35 BP**	1496 MYA
**16012A**	*Lasiorhinus krefftii*	Northern hairy-nosed wombat	QLD, Australia	Bullamon Station, Moonie River	Drilled bone powder, lower jaw	≈ 120	-
**16015A**	*Lasiorhinus krefftii*	Northern hairy-nosed wombat	QLD, Australia	Epping Forest Station	Drilled bone powder from leg bone	≈ 120	-
**16017A**	*Lasiorhinus krefftii*	Northern hairy-nosed wombat	QLD, Australia	Epping Forest Station	Fragments from nasal passage	≈ 120	-
**16019A**	*Lasiorhinus krefftii*	Northern hairy-nosed wombat	QLD, Australia	Epping Forest Station	Bone fragments nasal passage + proximate end teeth	≈ 120	-
**16024A**	*Lasiorhinus krefftii*	Northern hairy-nosed wombat	QLD, Australia	Epping Forest National Park	Drilled bone powder from leg bone	≈ 120	-
**16036A**	*Lasiorhinus krefftii*	Northern hairy-nosed wombat	QLD, Australia	Epping Forest National Park	Post-cranial, mostly vertebrae, drilled powder	≈ 120	-
**16037A**	*Lasiorhinus krefftii*	Northern hairy-nosed wombat	QLD, Australia	Epping Forest National Park	Drilled bone powder from leg bone	≈ 120	-
**16038A**	*Lasiorhinus krefftii*	Northern hairy-nosed wombat	QLD, Australia	Epping Forest National Park	Drilled bone powder from leg bone	≈ 120	-
**16039A**	*Lasiorhinus krefftii*	Northern hairy-nosed wombat	QLD, Australia	Epping Forest National Park	Drilled bone powder from leg bone	≈ 120	-
**16040A**	*Lasiorhinus krefftii*	Northern hairy-nosed wombat	QLD, Australia	Epping Forest National Park	Drilled bone powder from pelvis	≈ 120	-

Divergence times were generated with TimeTree (http://www.timetree.org) [19]. Calibrated carbon dates are given in bold. “≈” approximately, “-”not applicable

### Library construction and amplification

All library construction protocols were based on previously established methods for aDNA that included blunt end ligation of truncated Illumina adapters. The truncated adapters included internal barcodes and the protocols included an enzymatic treatment to partially remove uracils [15,20–22]. All taxa were taken through the *Bst* adapter fill-in stage of library construction and then amplified with different PCR protocols depending on the study from which the sample originated. Detailed descriptions of library construction and initial amplification are given in S1 Supplemental Methods.

### Shotgun library amplification (addition of full-length Illumina adapters)

A portion of each truncated library was converted into a sequencing library by amplifying with primers that completed the Illumina adapter structure [20,21]. The protocols for shotgun amplification are given in Methods section of the Supplemental Information.

### Mitochondrial DNA enrichment by hybridization capture

Each of the wombat DNA libraries was taken through a single round of hybridization capture using wombat probes and a wash procedure that followed the MyBaits User Manual Version 3.02 (http://www.arborbiosci.com/wp-content/uploads/2017/10/MYbaits-manual-v3.pdf). For the bison probes, two parallel hybridization capture reactions were performed for each non-wombat sample to evaluate the effect of high stringency washes (low salt, high temperature) and low stringency washes (high salt, low temperature) on enrichment efficiencies across different taxa. For high stringency washes we used the procedure outlined in the MyBaits User Manual and for the low stringency washes a protocol was used from a previous study involving our in-house probes [14].

Prior to hybridization capture, the following two solutions were prepared:

- Probe Solution: 9 μL 20x SSPE, 0.5 μL 0.5 M EDTA pH 8.0, 3.5 μL 50X Denhardt’s Solution, 0.5 μL 10% SDS, 1 μL SUPERase-In (20 U/μL, ThermoFisher), 125 ng in-house baits, 0.5 μL 50 μM RNA oligos to block truncated Illumina adapters [22], and nuclease-free H_2_O to 20 μL.
- Target Solution: 100 ng truncated Illumina library, 2.5 μL 1 μg/μL human Cot-1 DNA, 2.5 μL 1 μg/μL salmon sperm DNA, and nuclease-free H_2_O to 12 μL.

Hybridization capture was performed in a heated-lid thermal cycler. Tubes containing Target Solution were placed in the thermal cycler and incubated 5 min at 95°C. Tubes containing the Probes Solution were placed in the thermal cycler and incubated for 5 min at 65°C. Probe Solutions (18μL) were added to the Target Solutions and then mixed well by pipetting. The hybridization capture reactions were incubated for 5 hours at 65°C, 5 hours at 60° C, and ≈ 38 hours at 55°C (≈ 48 hours in total).

Just prior to the end of the incubations, MyOne Streptavidin C1 beads (ThermoFisher) were washed and non-specific binding sites blocked with yeast tRNA in preparation of recovering the annealed probe/target complexes. For each hybridization capture, 30 μL of beads were briefly washed with 500 μL binding buffer (1M NaCl, 10mM Tris-HCl, pH 7.5, 1mM EDTA) and then blocked by incubating the beads in 500 mL binding buffer + 10 μg yeast tRNA (ThermoFisher) for 30 min on a rotator at room temperature. After blocking, the beads were washed twice more with 500 μL binding buffer and then suspended in 70 μL binding buffer and placed in a 1.5 mL tube.

### High stringency washes (MyBaits protocol)

Blocked beads were warmed for 5 min in a heating block set to 55°C. Each hybridization capture reaction was added to an aliquot of blocked beads and then mixed by pipetting. These mixtures were incubated at 55°C for 30 min with the tubes flicked with fingers every 5 min to keep the beads in suspension. Afterwards, the beads were pelleted with a magnetic rack and the supernate discarded. Beads were then suspended in wash buffer (0.02X SSC + 0.1% SDS) warmed to 55°C and incubated at 55°C for 10 min with occasional flicking to resuspend the beads. These steps were repeated an additional two times for a total of three washes. Once the last wash supernate was discarded, the beads were suspended in 40 μL H_2_O and heated at 95° C for 5 min to release the DNA. The beads were precipitated with a magnetic rack and the released DNA was transferred to a new 1.5 ml tube for storage at -20° C.

### Low stringency washes

Each hybridization capture reaction was mixed with an aliquot of blocked beads at room temperature and then place on a rotator for 30 min at room temperature. Subsequently, the beads were pelleted with a magnetic rack, the supernate discarded, and the beads were taken through the following 4 washes: 0.5 mL 2x SSC + 0.05% Tween-20 at room temperature on a rotor for 10 min, 0.5 mL 0.75x SSC + 0.05% Tween-20 in a heating block at 50°C for 10 min, repeat previous wash, and 0.5 mL 0.20x SSC + 0.05% Tween-20 in a heating block at 50°C for 10 min. After the last wash the DNA was released from the beads as with the high stringency washes.

### Post-enrichment amplification

Each wombat mtDNA enriched library was amplified in 8 x 25 μL PCRs containing 2.5 μL 10x Gold PCR Buffer, 2 μL 2.5 mM dNTPs, 1.5 μL 25 mM MgCl_2,_ 0.5 μL each of 10 μM IS4 and indexing primers [21], 2 μL mtDNA enriched library, 0.125 μL AmpliTaq Gold DNA Polymerase (ThermoFisher), and molecular biology grade H_2_O to 25 μL. Amplification was performed in a heated-lid thermal cycler programed as follows: initial denaturation 95°C for 10 min; 18 cycles 95°C for 15 seconds, 60°C for 30 seconds, 72°C for 1 min; and final extension at 72°C for 5 min. PCRs from the same library were pooled and purified using Sera-Mag SpeedBeads.

The DNA recovered from hybridization capture with bison probes was quantified by qPCR [23] as before (see S1 Supplemental Methods). Recovered DNA was divided among 4 x 25 μL PCRs containing 2.5 μL 10x High Fidelity PCR Buffer, 1 μL 50 mM MgSO_4_, 0.5 μL 10 mM dNTPs, 0.5 μL each of 10 μM IS4 and indexing primers, 0.1 μL Platinum Taq DNA Polymerase High Fidelity (5 U/μL), and molecular biology grade H_2_O to 25 μL. The PCRs were amplified in a heated-lid thermal cycler programmed as follows: initial denaturation 94°C for 2 min, (see Table 3) cycles at 94°C for 15 sec, 58°C for 30 sec, 68°C for 45 sec, and a final extension at 68°C for 2 min. For the bison probe enrichments, the libraries processed with low and high stringency washes were amplified with primers containing different Illumina i7 indexes to allow separation of these samples during downstream bioinformatics analysis. PCRs from the same library were pooled and were purified using Sera-Mag SpeedBeads.

**Table 3:** PCR cycle number for indexing amplification of mtDNA enriched libraries (determined using qPCR).

	**Low Stringency Washes**	**High Stringency Washes**
**Bison**	20	26
**Bighorn sheep**	26	30
**Thylacine**	21	28
**Emu**	26	31
**Millet**	23	27

### High-throughput DNA sequencing

Indexed libraries were quantified on a TapeStation 4200 using a D1000 ScreenTape (Agilent) and sent to the Australian Genome Research Facility for sequencing on an Illumina NextSeq 500 platform. Wombat samples were sequenced with 2 x 75 bp paired-end (150 cycle) chemistry and all other libraries with 2 x 150 bp paired-end (300 cycle) chemistry.

### Data processing

**Fig 2:**
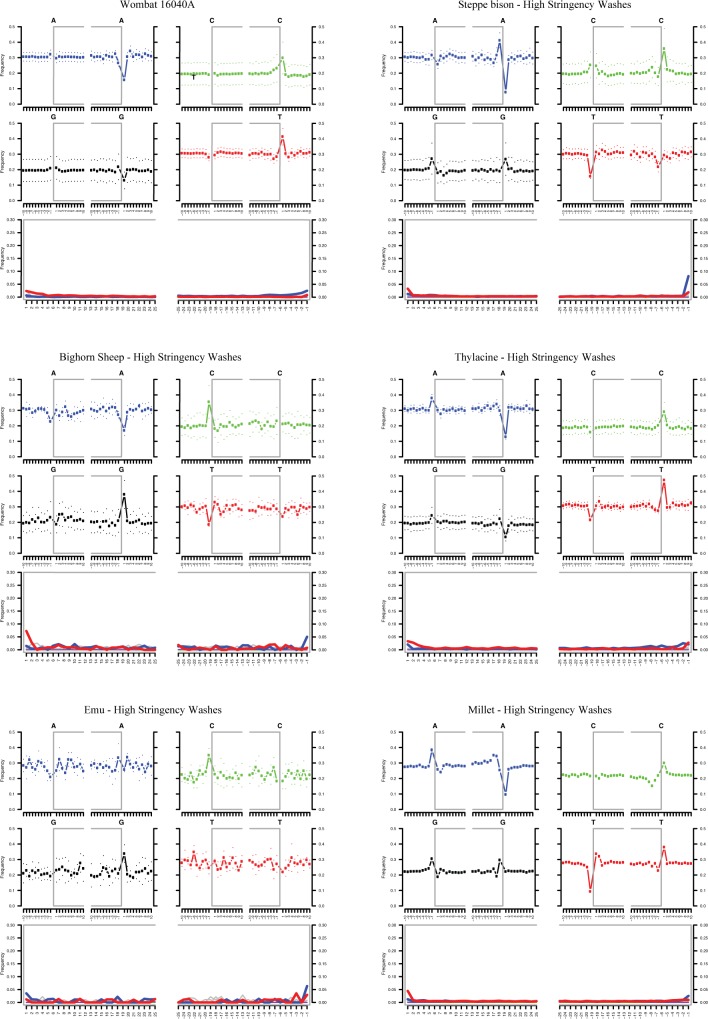
MapDamage plots. Levels of cytosine deamination in mitogenome mapped data were assayed with mapDamage 2 [27]. Only plots from hybridization capture enrichments that used high stringency washes are shown. Additionally, only a single northern hairy-nosed wombat sample is shown as an example. All samples exhibit increased levels of C → T and G → A substitutions that are typical of aDNA. Hybridization capture enrichments with low stringency washes and the other wombat samples gave similar results as those shown above.

The fastq files from the sequencer were demultiplexed by the internal barcodes using Sabre 1.0 (https://github.com/najoshi/sabre). AdapterRemoval v2.2.1 [24] was then used to trim adapters, collapse reads, discard reads < 25 bp, and remove reads of low quality (< 4). To eliminate the effect of sequencing depth and allow direct comparison of the washing conditions, all libraries enriched with bison probes were randomly subsampled to 2.5 x 10^6^ reads using the reformat command of BBTools (36.62-intel-2017.01: https://jgi.doe.gov/data-and-tools/bbtools/) and Java (1.8.0_121). The level of subsampling was determined by the total number of reads obtained from the library with the least reads. As hybridization capture conditions were not being compared for the wombat samples we did not subsample the reads from those libraries. Collapsed reads were mapped to mitochondrial reference genomes using BWA aln (v0.5.11-foss-2016b) [25,26]. Duplicate reads were removed from the mapped data using the SortSam and MarkDuplicates packages of Picard Tools (v 2.2.4: https://broadinstitute.github.io/picard/index.html). Read depths for the mtDNA enriched libraries were also generated using SAMtools (v1.3.1-foss-2016a). For the bison probe capture reactions, sequencing data from each species were mapped to a reference of the same species (i.e., emu reads were mapped to emu reference) except for the millet sample which was mapped to sorghum because currently there is no mitochondrial reference for broomcorn millet. The GenBank reference sequences used for mapping were: KJ868118.1 (wombat: nucleotides 1 to 15420), KX269121.1 (steppe bison: length = 16,320 bp), NC_015889.1 (bighorn sheep: length = 16,463 bp), FJ515781.1 (thylacine: length = 17,745 bp), AF338711.1 (emu: length = 16,711 bp), and NC_008360.1 (sorghum: length = 468,628 bp). Levels of nucleotide misincorporation caused by deaminated cytosine were assayed using mapDamage 2.0 [27]. The historic/ancient libraries enriched for mtDNA with in-house probes contained sequences that were fragmented (generally < 150 bp) and exhibited increased levels of deaminated cytosines at the ends of molecules indicative of authentic aDNA (Fig 2) [22]. Plots of mtDNA-enriched library read-depth with reference GC content were generated using R (v3.4.2).

## Results/Discussion

### Validation of protocol using wombat DNA

**Fig 3:**
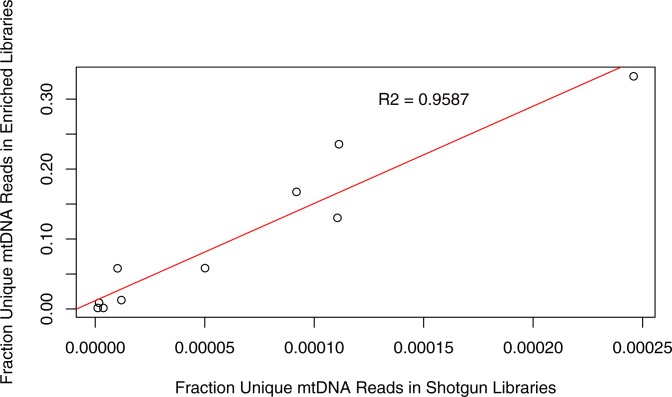
Correlation of fraction unique mapped reads in wombat libraries. The fraction of unique mapped reads for the wombat shotgun and mtDNA enriched libraries was generated by dividing the number of unique mapped reads by the number of collapsed reads (Table 4). The high correlation between the fraction of unique reads in shotgun and enriched libraries suggest that initial screening of a sample with shallow sequencing and adjusting sequencing effort accordingly may reduce the cost of studies involving hybridization capture enrichment.

We validated our protocol by applying it to sequence mtDNA from museum specimens of the northern hairy-nosed wombat. Of the 10 hairy-nosed wombat historic specimens, one (16036A) contained very few mitochondrial sequences in the pre and post-enrichment libraries and was likely too poorly preserved for study. For the remaining 9 wombat samples, hybridization capture with our in-house probes produced a 28 to 264 fold enrichment (average = 139; standard deviation = 89) of mtDNA sequences and generated mean unique read depths that were on average >111 times greater than shotgun libraries (Table 4). For comparison, a study that used commercial enrichment probes on camel aDNA reported fold enrichments of mtDNA that ranged from 0.2 to 1,153 with an average of 187 [28]. The endogenous DNA proportions of the wombat samples, as judged by the fraction of unique mtDNA reads in shotgun libraries was strongly correlated with unique mitochondrial sequences in the final enriched libraries (Fig 3).

**Table 4:** Wombat mapping statistics.

**Sample**	**Collapsed Reads**	**Mapped Reads**	**Unique Mapped** **Reads**	**Minimum Unique Read Depth**	**Maximum Unique Read Depth**	**Mean Unique Read Depth**	**% Reference Covered by at Least Unique** **One Read**	**Fold Enrichment** **Of Unique Reads**	**Fold Increase in Mean Unique Read Depth**
**Shotgun Libraries**									
**16012A**	4219884	43	43	0	2	0.23	20.93	-	-
**16015A**	1755334	88	86	0	4	0.54	39.94	-	-
**16017A**	2993544	11	11	0	2	0.07	6.61	-	-
**16019A**	5445759	9	9	0	1	0.05	4.71	-	-
**16024A**	1446171	160	157	0	8	0.95	55.19	-	-
**16036A**	2790106	3	3	0	3	0.01	0.54	-	-
**16037A**	2339861	215	214	0	8	1.17	64.49	-	-
**16038A**	3627332	43	43	0	3	0.25	20.82	-	-
**16039A**	2533495	282	276	0	7	1.60	78.95	-	-
**16040A**	2907911	715	691	0	14	4.01	96.13	-	-
**mtDNA Enriched Libraries**									
**16012A**	4940987	286958	7030	0	57	34.83	99.96	139.63	151.43
**16015A**	316534	18500	3098	0	51	19.13	99.87	199.77	35.43
**16017A**	1083154	1522	943	0	21	5.28	97.80	236.93	75.43
**16019A**	4774242	41996	1945	0	32	10.24	99.13	246.51	204.8
**16024A**	4137981	538950	12843	0	153	85.07	99.99	28.59	89.55
**16036A**	3626269	5173	82	0	73	0.33	1.44	21.03	84.53
**16037A**	3449102	577350	15649	1	159	98.90	100.00	49.61	249.84
**16038A**	3952352	49720	10372	0	141	62.46	99.99	221.37	61.9
**16039A**	1396352	328930	15426	0	161	99.04	99.98	101.41	49.97
**16040A**	3428373	1139995	27564	6	239	200.36	100.00	33.83	151.43

Ancient DNA from museum hairy-nosed northern wombat specimens was converted into sequencing libraries and enriched for mtDNA. Shotgun and mtDNA enriched libraries were mapped to a truncated wombat mitogenome reference (GenBank: KJ868118.1, bp 1 to 15420) using BWA aln [25].

### Hybridization capture of targets from phylogenetically distant taxa

The primary goal of this study was to determine if probes produced from modern bison DNA could be used to successfully enrich for mtDNA from phylogenetically divergent taxa. The ability to perform cross-taxon enrichment would demonstrate that our system is flexible and that one set of in-house probes could be used to capture target DNA from multiple taxa. While successful hybridization capture of taxa divergent from probe sequences has been reported previously [11–13], these studies used commercial probes of a standard length in contrast to our randomly fragmented probes of variable length [14]. Further we also sought to examine the impact of low and high stringency washes on the enrichment of targets from divergent taxa.

We transcribed probes from modern bison (American and European) and then enriched mtDNA from species that ranged from close relatives such as steppe bison, to very distant species such as broomcorn millet (with an estimated divergence time from modern bison of ≈ 1.5 billion years ago [19]). As expected, probes synthesized from modern bison were readily able to enrich mtDNA from steppe bison libraries (Table 5). Compared to the other taxa examined, the steppe bison sample underwent the greatest enrichment of unique reads (> 55 fold increase) and greatest coverage of the reference genome (> 99.8% of the reference covered by at least 1 unique read) with the bison probes. The bison probes were able to enrich sequences from divergent animal taxa, producing 2.8 to 61.55-fold enrichment of unique reads for the bighorn sheep, thylacine, and emu libraries. Enrichment with bison probes increased the percentage of the mitochondrial reference being covered by unique reads for the bighorn sheep (17.71% to 97.25%) and emu (10.25% to 70.7%) samples, but in contrast produced only a slight (<1%) change in the thylacine (Table 5).

**Table 5:** Mapping statistics for subsampled divergent taxa libraries.

**Sample**	**Mapped Reads**	**Unique Mapped Reads**	**Minimum Unique Read Depth**	**Maximum Unique Read Depth**	**Mean Unique Read Depth**	**% of Reference Covered by at Least 1 Unique Read**	**Fold Increase Unique reads**	**Fold Increase in Mean Unique Read Depth**
**Shotgun Libraries**								
**Bison**	144	143	0	5	0.97	63.36	-	-
**Bighorn sheep**	57	56	0	3	0.19	17.71	-	-
**Thylacine**	3714	3327	0	57	14.37	93.09	-	-
**Emu**	26	26	0	3	0.11	10.25	-	-
**Millet**	35500	32138	0	82	5.26	39.50	-	-
**mtDNA Enriched Libraries–Low Stringency Washes**								
**Bison**	13408	7891	0	202	60.96	99.89	55.18	62.85
**Bighorn sheep**	65992	3447	0	42	13.20	97.25	61.55	69.47
**Thylacine**	181291	11171	0	310	70.39	94.04	3.36	4.90
**Emu**	5943	1231	0	75	8.10	70.70	47.35	73.64
**Millet**	30790	24969	0	204	4.12	38.86	0.78	0.78
**mtDNA Enriched Libraries–High Stringency Washes**								
**Bison**	71992	15320	1	339	144.92	100.00	107.13	149.40
**Bighorn sheep**	27076	1057	0	32	4.20	82.17	18.88	22.11
**Thylacine**	481887	9306	0	335	58.14	93.06	2.80	4.05
**Emu**	7634	339	0	69	2.45	42.04	13.04	22.27
**Millet**	34030	21829	0	99	3.55	38.85	0.68	0.67

In-house hybridization capture probes made from modern bison DNA were used to enrich mtDNA from ancient specimens of divergent taxa. To eliminate the effect of sequencing depth, analysis was performed on 2.5 x 10^6^ reads randomly subsampled from each library. The bison probes were able to enrich divergent animal mtDNA but not plant. The effect of low stringency washes (low temperature and high salt concentration) and high stringency washes (high temperature and low salt concentration) on enrichment efficiency with in-house bison probes indicates that high stringency washes are more effective with closely related taxa while low stringency washes are more effective with distantly related species. Mapping statistics for the entire sequencing data set are given in S2 Table.

The bison probes were ineffective at enriching millet mtDNA, as expected given the evolutionary distance and sequence divergence involved. The inability of our in-house bison probes to enrich highly divergent millet mtDNA provides a negative control for the methodology and demonstrates that the mtDNA enrichment is dependent on the interaction of our probe with target sequences and is not the result of non-specific activity that favouring mitochondrial over nuclear molecules. Shotgun data indicate that mitochondrial sequences are present in the millet library and that the lack of enrichment is not due the sample being too degraded for recovery of mtDNA.

### Wash stringency

High stringency washes encourage the denaturation of annealed nucleic acids so that only probes/library complexes with high levels of sequence homology remained bound together contrast to low stringency washes. The impact of wash stringency on the recovery of target DNA is demonstrated by the additional 4 to 6 cycles needed (determined by qPCR) to amplify the libraries that underwent high stringency washes (Table 3). As expected, the high stringency washes proved more effective at enriching steppe bison sequences than the low stringency washes (107.13 versus 55.18 fold enrichment of unique reads, Table 5). In contrast, the low stringency washes proved to be more effective with all the other animal samples. The sequence divergence between these species and modern bison would make probe/library complexes less stable because of sequence mismatches so the less disruptive nature of the low stringency washes allowed the recovery of more target sequences.

### GC content

**Fig 4:**
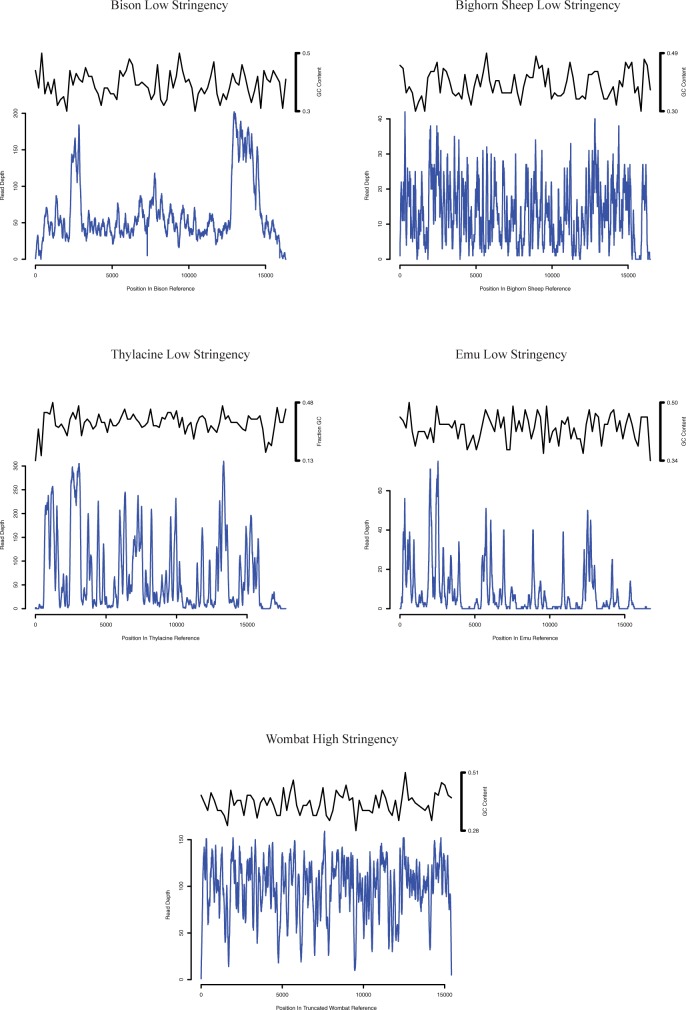
Read depth of animal mtDNA enriched libraries. Read depths of animal sequencing libraries enriched for mtDNA and mapped to a mitogenome reference. The small black graph above the read depth plot represents the fraction of GC nucleotides in reference mitogenome in a 200 bp sliding window. Sequence composition did not appear to have a consistent impact on the recovery of mtDNA with the RNA probes. Some regions of references with low GC content did appear to have reduced levels of read depth, which include nucleotides 16,151 to 16,743 of the thylacine reference where the GC content drops to ≈ 13%. The large variability in GC content of the thylacine mitogenome may have contributed to the reduced enrichment efficiency observed with the marsupial sample.

The nucleotide content of enriched mitogenomes did not appear to have a consistent impact on the recovery of mtDNA in most of the animal samples (Fig 4). Some drops in GC content of the wombat mitogenome appear to be associated with reduced read depth, but this pattern was not universal. In a segment of the thylacine mitogenome (bp 16,151 to 16,743) the GC content is only 13% and this locus appears to be completely devoid of mapped reads. The reduced data in these low GC content areas likely stems from several factors: PCR amplification is biased against low GC content sequences [29] and hybridization capture methods generally recover sequences with extreme GC content (low and high) less efficiently [30]. Similar to other hybridization capture methods, our in-house probes do not enrich loci with extreme GC content efficiently and this limitation must be considered when designing a study.

The thylacine mtDNA enrichments did not perform in the same manner as the other animals and produced only ≈ 3 fold enrichment compared to the >13 increase of observed for the animals (Table 5). The combination of GC content and phylogenetic distance may have contributed to lower performance of the bison probes with the thylacine sample. The GC content of the thylacine mitogenome spans 35 percentage points in comparison to the maximum of 23 observed in the other animal mitogenomes (Fig 4). This broad range, which includes regions of very low GC content, coupled with the sequence divergence between thylacine and bison mitogenomes may have reduced the efficiency of the in-house probes with the marsupial sample.

### Probe cost

The estimated the cost of our in-house mitochondrial probes for a single hybridization was approximately $7.00 (USD). Similar mitochondrial probes from a commercial source would cost approximately $8.00 to $18.00 (USD) depending on the number of reactions purchased. Although the cost of commercial reagents can approach the price of our in-house probes this would require the purchase of a kit that is sufficient to enrich several hundred mitogenomes, which may not be suitable for all studies. Our methodology did require an initial expenditure for various reagents and kits, but after this purchase probes were produced inexpensively.

### Advantages over comparable protocols

Previously, Maricic et al. (2010) described a probe synthesis procedure somewhat similar to the methodology described in the current paper [5]. In Maricic et al., probes were synthesized by attaching biotin directly to fragmented long-range amplicons instead of transcribing RNA from these PCR products. The procedure described in the current study has several advantages over the Maricic et al. (2010) protocol. Firstly, the *in vitro* transcription of the long-range amplicons results in an amplification of RNA molecules and increased probe yield [31]. Second, only single stranded RNA is transcribed in our methodology [31], so there will be minimal formation of probe/probe complexes which is a concern for the probe synthesized from dsDNA using methods such as the Maricic et al. (2010) protocol.

### Conclusion

In this study, we have described a method to synthesize hybridization capture probes from overlapping long-range PCR amplicons, which has substantial advantages over comparable in-house protocols. Our procedure does not require any specialized instruments and the probes can be synthesized in most standardly equipped molecular biology laboratories. The methodology is flexible as researchers can design primers with the T7 promoter to enrich targets as needed. Target size should also be flexible in our system, as primer sets can be designed to enrich small to modest sized targets including mitogenomes, chloroplast genomes, exons, or any genomic region spanning a few tens of kilobases. However, enriching a large target such as a complete exome is not feasible with our system, as the effort needed to produce the long-range templates would make commercial probes more cost effective.

Our probe synthesis method offers a less expensive and more flexible alternative to commercial reagents. In the current study, we demonstrate that our probes behave in a consistent manner and will even enrich mitochondrial genomes from ancient samples with damaged DNA. Importantly, we also demonstrate that our in-house system can be used to enrich target DNA from taxa that are phylogenetically distant from the species used to transcribe the probe molecules, especially when the stringency of the wash steps is lowered (by increasing salt concentration and lowering temperature). This should remove the need for phylogenetically diverse probe sets, and make in-house probe synthesis viable and cost-effective even for small projects on varied taxa.

## Supporting information

S1 Table Additional sample information. (DOCX)

S2 Table Mapping statistics of complete sequencing data. (DOCX)

S1 Supplemental Methods (DOCX)
