# Correction: Low-cost cross-taxon enrichment of mitochondrial DNA using in-house synthesised RNA probes

**DOI:** 10.1371/journal.pone.0213296

**Published:** 2019-02-27

**Authors:** Stephen M. Richards, Nelli Hovhannisyan, Matthew Gilliham, Joshua Ingram, Birgitte Skadhauge, Holly Heiniger, Bastien Llamas, Kieren J. Mitchell, Julie Meachen, Geoffrey B. Fincher, Jeremy J. Austin, Alan Cooper

In the Funding statement, the following grant numbers are missing from the Carlsberg Foundation: CF15-0672 and CF16-0551.
