# Supplementary material for: Low-cost cross-taxon enrichment of mitochondrial DNA using in-house synthesised RNA probes
Source: PLoS One. 2019 Feb 4;14(2):e0209499. doi: 10.1371/journal.pone.0209499 (PMC6361428; doi:10.1371/journal.pone.0209499)
Supplement: S1 Table — (DOCX) [file pone.0209499.s001.docx]

**S1 Table. Additional Sample Information**

| ACAD/study identifier | Common Name | Publicly available | Country of origin | Holding institution | Holding institution identifier | Permit for import into Australia | Permit for research |
| --- | --- | --- | --- | --- | --- | --- | --- |
|  |  |  |  |  |  |  |  |
| 16012A | Northern hairy nosed wombat | Yes | Australia | Queensland Museum | J13145 | Not applicable | None Required by country of origin |
| 16015A | Northern hairy nosed wombat | Yes | Australia | Queensland Museum | J20354 | Not applicable | None Required by country of origin |
| 16017A | Northern hairy nosed wombat | Yes | Australia | Queensland Museum | J6240 | Not applicable | None Required by country of origin |
| 16019A | Northern hairy nosed wombat | Yes | Australia | Queensland Museum | J6284 | Not applicable | None Required by country of origin |
| 16024A | Northern hairy nosed wombat | Yes | Australia | Queensland Museum | JM5251 | Not applicable | None Required by country of origin |
| 16036A | Northern hairy nosed wombat | Yes | Australia | Queensland Museum | JM8462 | Not applicable | None Required by country of origin |
| 16037A | Northern hairy nosed wombat | Yes | Australia | Queensland Museum | JM8463 | Not applicable | None Required by country of origin |
| 16038A | Northern hairy nosed wombat | Yes | Australia | Queensland Museum | JM8464 | Not applicable | None Required by country of origin |
| 16039A | Northern hairy nosed wombat | Yes | Australia | Queensland Museum | JM8465 | Not applicable | None Required by country of origin |
| 16040A | Northern hairy nosed wombat | Yes | Australia | Queensland Museum | JM8466 | Not applicable | None Required by country of origin |
|  |  |  |  |  |  |  |  |
| 3133A | Steppe bison | Yes | Canada | Australian Centre for Ancient DNA, University of Adelaide | 3133 | Imported into Australia under Permit to Import Quarantine Material 200603672 in accordance with Australian Government Quarantine Act 1908 Section 13(2AA) | None Required by country of origin |
| 18154A | Bighorn Sheep | Yes | USA | University of Wyoming Geological Museum, USA | UW52695 | Imported into Australia under Permit to Import Quarantine Material IP15007179 (entry number (NA15086074) in accordance with Australian Government Quarantine Act 1908 Section 13(2AA) | Permission for research on this sample was granted by the Bureau of Land Management as part of U.S. National Science Foundation project EAR-SGP #1425059 |
| 18220A | Enu | Yes | Australia | Queen Victoria Museum and Art Gallery, Australia | QVM:2016:2:0008 | Not applicable | None Required by country of origin |
| 9743A | Thylacine | Yes | Australia | Tasmanian Museum and Art Gallery | A321 | Not applicable | None Required by country of origin |
| 112295a | Broomcorn millet | No (Destroyed for extraction of ancient DNA) | Armenia | Australian Centre for Ancient DNA, University of Adelaide | 112295 | Imported into Australia under Permit to Import Quarantine Material IP11009972 (entry number (SA11011701) in accordance with Australian Government Quarantine Act 1908 Section 13(2AA) | None Required by country of origin |
