# Supplementary material for: Low-cost cross-taxon enrichment of mitochondrial DNA using in-house synthesised RNA probes
Source: PLoS One. 2019 Feb 4;14(2):e0209499. doi: 10.1371/journal.pone.0209499 (PMC6361428; doi:10.1371/journal.pone.0209499)
Supplement: S2 Table — (DOCX) [file pone.0209499.s002.docx]

**S2 Table. Mapping Statistics of Complete Sequencing Data**

| Sample | Collapsed Reads | Mapped Reads | Unique Mapped Reads | Minimum Unique Read Depth | Maximum Unique Read Depth | Mean Unique Read Depth | % Reference Covered by at least 1 Unique Read | Fold Enrichment of Unique Reads | Fold Increase In Mean Read Depth |
| --- | --- | --- | --- | --- | --- | --- | --- | --- | --- |
|  |  |  |  |  |  |  |  |  |  |
| Shotgun Libraries | | | | | | | | | |
|  |  |  |  |  |  |  |  |  |  |
| Bison | 4188819 | 239 | 234 | 0 | 6 | 1.57 | 80.56 | - | - |
| Bighorn Sheep | 3222657 | 76 | 75 | 0 | 3 | 0.26 | 22.88 | - | - |
| Thylacine | 3442070 | 5713 | 4437 | 0 | 63 | 19.31 | 93.27 | - | - |
| Emu | 3435927 | 38 | 38 | 0 | 3 | 0.17 | 15.27 | - | - |
| Millet | 3190155 | 45269 | 39929 | 0 | 90 | 6.55 | 39.7 | - | - |
|  |  |  |  |  |  |  |  |  |  |
| mtDNA Enriched Libraries with Low Stringency Washes | | | | | | | | | |
|  |  |  |  |  |  |  |  |  |  |
| Bison | 7884181 | 42611 | 15037 | 0 | 278 | 123.57 | 99.94 | 34.14 | 78.71 |
| Bighorn Sheep | 5029794 | 132633 | 3766 | 0 | 42 | 14.35 | 97.98 | 32.17 | 55.19 |
| Thylacine | 6104866 | 443080 | 15027 | 0 | 333 | 98.61 | 94.17 | 1.91 | 5.11 |
| Emu | 6166413 | 14565 | 1680 | 0 | 80 | 11.66 | 80.09 | 24.63 | 68.59 |
| Millet | 5068737 | 62353 | 45281 | 0 | 223 | 7.52 | 39.71 | 0.71 | 1.15 |
|  |  |  |  |  |  |  |  |  |  |
| mtDNA Enriched Libraries with High Stringency Washes | | | | | | | | | |
|  |  |  |  |  |  |  |  |  |  |
| Bison | 3312395 | 95060 | 16695 | 1 | 345 | 160.82 | 100 | 90.22 | 102.43 |
| Bighorn Sheep | 2718756 | 29374 | 1060 | 0 | 32 | 4.21 | 82.17 | 16.75 | 16.19 |
| Thylacine | 2892797 | 557722 | 9472 | 0 | 358 | 59.57 | 93.11 | 2.54 | 3.08 |
| Emu | 4453254 | 13653 | 360 | 0 | 70 | 2.67 | 42.57 | 7.31 | 15.71 |
| Millet | 3161357 | 43221 | 25100 | 0 | 110 | 4.1 | 39.2 | 0.63 | 0.63 |
