# Supplementary material for: Low-cost cross-taxon enrichment of mitochondrial DNA using in-house synthesised RNA probes
Source: PLoS One. 2019 Feb 4;14(2):e0209499. doi: 10.1371/journal.pone.0209499 (PMC6361428; doi:10.1371/journal.pone.0209499)
Supplement: S1 Supplemental Methods — (DOCX) [file pone.0209499.s003.docx]

Low-cost cross-taxon enrichment of mitochondrial DNA using in-house synthesised RNA probes

Stephen M. Richards^1^, Nelli Hovhannisyan^2^, Matthew Gilliham^3^, Joshua Ingram^1^, Birgitte Skadhauge^4^, Holly Heiniger^1^, Bastien Llamas^1^, Kieren J. Mitchell^1^, Julie Meachen^5^, Geoffrey B. Fincher^6^, Jeremy J. Austin^1^,

Alan Cooper^1^

1 Australian Centre for Ancient DNA, University of Adelaide, Adelaide, Australia

2 Yerevan State University, Yerevan, Armenia

3 ARC Centre of Excellence in Plant Energy Biology, Waite Research Institute, University of Adelaide, Adelaide, Australia

4 Carlsberg Research Laboratory, J.C. Jacobsens Gade 10 DK1799, Copenhagen, Denmark

5 Des Moines University, Des Moines, United States of America

6 ARC Centre of Excellence in Cell Walls, Waite Research Institute, University of Adelaide, Adelaide, Australia

Supplemental Information

**Methods**

*Extraction of aDNA*

Ancient DNA extractions and sequencing library construction were performed in dedicated low-DNA laboratories physically separated from post-amplification workspace. Standard guidelines for working with aDNA were followed including extraction blanks for all samples and negative controls for all PCRs[1].

Animal samples: All historical/ancient animal samples were extracted with a published in-solution silica protocol [2], except for the thylacine which was extracted with a modified silica method [2, 3].

Historical broomcorn millet: A millet seed was washed for 3 minutes with 100 µL EB Buffer (Qiagen) + 0.05% Tween and then 100 µL 100% ethanol in a shaking heating block set to 25°C and 500 RPM. The seed was then placed in a screw cap tube containing 5 Zirconia/Silica beads (2.3 mm, Biospec) and then ground to powder by agitating the tube in a FastPrep at 6.5 meters/second for 45 seconds. To extract DNA, 150 µL Plant DNazol (ThermoFisher) + 2% PVP (Sigma-Aldrich) was added and the resulting mixture shaken in a thermomixer for 5 minutes (500 RPM) at room temperature. An equal volume of chloroform (150 µL) was added and the tube was subsequently vigorously shaken for 5 minutes and incubated on a bench top for 15 min. The liquid was then transferred to a fresh 1.7 mL tube and centrifuged at 12,000 g for 10 minutes. The supernate was removed and further purified with MinElute spin columns (Qiagen) following the manufacturer’s instructions except the final elution was performed with 22 µL EB buffer (Qiagen) + 0.05% Tween-20.

*Initial library amplification*

Wombat and Thylacine: Forty µL of the *Bst* reaction was divided among 8 x 25 µL PCRs containing 2.5 µL 10x High Fidelity PCR Buffer, 1 µL 50 mM MgSO_4_, 0.2 µL 25 mM dNTPs, 0.5 µL each of 10 µM IS7 and IS8 primers [4], 0.1 µL Platinum Taq DNA Polymerase High Fidelity (ThermoFisher: 5 U/μL), and molecular biology grade H_2_O to 25 µL. The PCRs were amplified in a heated-lid thermal cycler programmed as follows: initial denaturation 94ºC for 2 min, 13 cycles at 94ºC for 30 sec, 60ºC for 30 sec, 68ºC for 40 sec, and a final extension at 68ºC for 2 min.

Bison: Forty µL of the *Bst* reaction was divided among 8 x 25 µL PCRs containing 2.5 µL 10x High Fidelity PCR Buffer, 1 µL 50 mM MgSO_4_, 0.2 µL 25 mM dNTPs, 0.5 µL each of 10 µM IS7 and IS8 primers, 0.1 µL Platinum Taq DNA Polymerase High Fidelity (5 U/μL), and molecular biology grade H_2_O to 25 µL. The PCRs were amplified in a heated-lid thermal cycler programmed as follows: initial denaturation 94ºC for 2 min, 9 cycles at 94ºC for 15 sec, 58ºC for 30 sec, 68ºC for 45 sec, and a final extension at 68ºC for 2 min. Library was purified with 1.8 volumes Sera-Mag SpeedBeads (GE Healthcare) [5] and eluted with 30 µL EB buffer + 0.05% Tween-20. One µL of this elution was quantified using qPCR to determine the minimum number of PCR cycles to amplify this library to produce sufficient DNA for downstream procedures [6]. Further amplification of the bison library was performed in 8 x 25 µL PCRs containing 2.5 µL 10x High Fidelity PCR Buffer, 1 µL 50 mM MgSO_4_, 0.2 µL 25 mM dNTPs, 0.5 µL each of 10 µM IS7 and IS8 primers, 0.1 µL Platinum Taq DNA Polymerase High Fidelity (5 U/μL), 3 µL eluted library, and molecular biology grade H_2_O to 25 µL. The PCRs were amplified in a heated-lid thermal cycler programmed as follows: initial denaturation 94ºC for 2 min, 8 cycles at 94ºC for 15 sec, 58ºC for 30 sec, 68ºC for 45 sec, and a final extension at 68ºC for 2 min. The library from this second amplification was used in all subsequent steps.

Bighorn sheep and emu: One µL of the *Bst* reaction was quantified with qPCR as before to determine the appropriate cycle number to produce sufficient library for downstream procedures [6]. After quantification, the remaining 39 µL of the *Bst* reaction was divided among 8 x 25 µL PCRs containing 2.5 µL 10x High Fidelity PCR Buffer, 1 µL 50 mM MgSO_4_, 0.25 µL 25 mM dNTPs, 0.5 µL each of 10 µM IS7 and IS8 primers, 0.1 µL Platinum Taq DNA Polymerase High Fidelity (5 U/μL), and molecular biology grade H_2_O to 25 µL. The PCRs were amplified in a heated-lid thermal cycler programmed as follows: initial denaturation 94ºC for 6 min, 13 or 15

cycles (for bighorn sheep and emu respectively) at 94ºC for 30 sec, 60ºC for 30 sec, 72ºC for 40 sec, and a final extension at 72ºC for 10 min.

Millet: Forty microliters of the library *Bst* reaction was divided among 8 x 25 µL PCRs containing 2.5 µL 10x High Fidelity PCR Buffer, 1 µL 50 mM MgSO_4_, 0.5 µL 10 mM dNTPs, 0.5 µL each of 10 µM IS7 and IS8 primers [4], 0.1 µL Platinum Taq DNA Polymerase High Fidelity (5 U/μL), and molecular biology grade H_2_O to 25 µL. The PCRs were amplified in a heated-lid thermal cycler programmed as follows: initial denaturation 94ºC for 2 min, 15 cycles at 94ºC for 15 sec, 58ºC for 30 sec, 68ºC for 45 sec 40, and a final extension at 68ºC for 2 min.

For all samples, PCRs from the same library were pooled and purified with 1.8 volumes Sera-Mag SpeedBeads [5] and quantified with a Qubit broad range dsDNA assay (ThermoFisher). Purified libraries were visualized with gel electrophoresis (2% agarose) and GelRed (Biotium) staining.

*Shotgun Library Amplification (addition of full length Illumina adapters)*

Wombat libraries were diluted to 1 ng/µL in EB + 0.5% Tween. Five µL of diluted library from the 10 wombat samples used in this study and 5 µL from each of 14 samples not used in the current study were pooled (total volume = 120 µL). The library pool was then amplified in 8 x 25 µL PCRs containing 2.5 µL 10x Gold PCR Buffer, 2 µL 2.5 mM dNTPs, 1.5 µL 25 mM MgCl_2,_ 0.5 µL each of 10 µM IS4 and indexing primers [4], 15 µL diluted library pool, 0.125 µL AmpliTaq Gold DNA Polymerase, and molecular biology grade H_2_O to 25 µL. Amplification was performed in a heated-lid thermal cycler programed as follows: initial denaturation 95ºC for 10 min; 4 cycles 95ºC for 15 sec, 60ºC for 30 sec, 72ºC for 1 min; and final extension at 72ºC for 5 min.

For non-wombat samples, 30 ng of each initial library amplification was divided among 4 x 25 µL PCRs containing 2.5 µL 10x High Fidelity PCR Buffer, 1 µL 50 mM MgSO_4_, 0.5 µL 10 mM dNTPs, 0.5 µL each of 10 µM IS4 and indexing primers, 0.1 µL Platinum Taq DNA Polymerase High Fidelity (5 U/μL), and molecular biology grade H_2_O to 25 µL. The PCRs were amplified in a heated-lid thermal cycler programmed as follows: initial denaturation 94ºC for 2 min, 6 cycles at 94ºC for 15 sec, 58ºC for 30 sec, 68ºC for 45 sec, and a final extension at 68ºC for 2 min. The library was purified with 1.8 volumes Sera-Mag SpeedBeads [5] and quantified with a Qubit broad range dsDNA assay (ThermoFisher).

References

1. Cooper A, Poinar HN. Ancient DNA: Do it right or not at ALL. Science. 2000;289(5482):1139-. PubMed PMID: ISI:000088866600013.

2. Brotherton P, Haak W, Templeton J, Brandt G, Soubrier J, Jane Adler C, et al. Neolithic mitochondrial haplogroup H genomes and the genetic origins of Europeans. Nature communications. 2013;4:1764. doi: 10.1038/ncomms2656.

3. White LC, Mitchell KJ, Austin JJ. Ancient mitochondrial genomes reveal the demographic history and phylogeography of the extinct, enigmatic thylacine (Thylacinus cynocephalus). J Biogeogr. 2017:n/a-n/a. doi: 10.1111/jbi.13101.

4. Meyer M, Kircher M. Illumina Sequencing Library Preparation for Highly Multiplexed Target Capture and Sequencing. Cold Spring Harbor Protocols. 2010;2010(6):pdb.prot5448. doi: 10.1101/pdb.prot5448.

5. Rohland N, Reich D. Cost-effective, high-throughput DNA sequencing libraries for multiplexed target capture. Genome Research. 2012;22(5):939-46.

6. Carøe C, Gopalakrishnan S, Vinner L, Mak SST, Sinding MHS, Samaniego JA, et al. Single-tube library preparation for degraded DNA. Methods in Ecology and Evolution. 2017:n/a-n/a. doi: 10.1111/2041-210X.12871.
